# *Achromobacter xylosoxidans* as a new microorganism strain colonizing high-density polyethylene as a key step to its biodegradation

**DOI:** 10.1007/s11356-016-6563-y

**Published:** 2016-04-13

**Authors:** Anna Kowalczyk, Marek Chyc, Przemysław Ryszka, Dariusz Latowski

**Affiliations:** 1 Department of Plant Physiology and Biochemistry, Faculty of Biochemistry, Biophysics and Biotechnology, Jagiellonian University, Gronostajowa 7, 30-387 Krakow, Poland; 2 Department of Environmental Protection, Mickiewicza 8, 33-100 Tarnow, Poland; 3 Faculty of Biology and Earth Sciences, Institute of Environmental Sciences, Gronostajowa 7, 30-387 Krakow, Poland; 4 Department of Environment Protection, Faculty of Geology, Geophysics and Environment Protection, University of Science and Technology, Mickiewicza 30, 30-059 Krakow, Poland

**Keywords:** Polymers, HDPE, Biodegradation, Microorganisms, FTIR, SEM

## Abstract

This study presents results of research on isolation new bacteria strain *Achromobacter xylosoxidans* able to effect on the structure of high-density polyethylene (HDPE), polymer resistant to degradation in environment. New strain of *A. xylosoxidans* PE-1 was isolated from the soil and identified by analysis of the 16S ribosome subunit coding sequences. The substance to be degraded was HDPE in the form of thin foil films. The foil samples were analyzed with Attenuated Total Reflectance Fourier Transform Infrared Spectroscopy (ATR-FTIR) as well as scanning electron microscope (SEM), and the results revealed degradation of chemical structure of HDPE. About 9 % loss of weight was also detected as a result of *A. xylosoxidans* PE-1 effect on HDPE foil. On the basis of comparative spectral analysis of the raw material before the bacteria treatment and the spectrum from a spectra database, it was assumed that the HDPE was the only source of carbon and energy for the microorganisms. No fillers or other additives used in the plastic processing were observed in HDPE before experiments. This is the first communication showing that *A. xylosoxidans* is able to modify chemical structure of HDPE, what was observed both on FTIR, in mass reduction of HDPE and SEM analysis. We also observed quite good growth of the bacteria also when the HDPE was the sole carbon source in the medium. These results prove that *A. xylosoxidans* is an organism worth applying in future HDPE biodegradation studies.

## Introduction

Disposal of waste is one of the most important current issues of environmental protection. Plastic waste, containing high-density polyethylene, is strongly resistant to degradation (Chiellini *et al.* 2003; Arutchelvi *et al*. 2008 *;* Borghei *et al*. 2010). Used HDPE is disposed to landfill, usually burned, which leads to contamination of the air with toxic compound, such as polycyclic hydrocarbons (Scott 2003). Less frequently, the HDPE is recycled, which is still an expensive method of utilization. The ability of biodegradation of PE by microorganisms is tested since the 70s, under field as well as laboratory conditions. The natural environment of organisms potentially able to damage the chemical and physical structure of such polymer is usually associated with contamination or waste treatment, e.g., soil, waters, sewage treatment plants, or another areas of waste disposal harboring a variety of micro- and macroorganisms (Yamada-Onodera et al. 2001; Gilan *et al*. 2004; Hadad *et al*. 2005; Immanuel *et al*. 2014; Yang *et al*. 2014).

The main objective of this study was to screen microorganisms not tested in research on biodegradation as far, which are able to change the structure of HDPE, using it as a carbon source for the metabolic processes and then demonstrate changes that occur both in the chemical structure and physical properties of the polymer as a result of their activity. The culture conditions were optimized to increase the capacity and efficiency of microbial proliferation, and selected strain of microorganisms was identified.

## Materials and methods

Microbial cultures were cultivated in liquid nutrient medium devoid of carbon. The source of this element for the microorganisms was sectioned HDPE polyethylene foil, the polymeric material comprising in its chemical structure only carbon and hydrogen in the form of unbranched hydrocarbon chain. The biological material was cultivated on solidified Luria-Bertani Broth (LB) medium.

### Microorganisms

In order to obtain microbial material, samples of polyethylene bags from illegal waste sites were collected. Samples were collected directly from the soil and were pieces of polyethylene shopping bags littering the landfill along the Mleczna River in Radom, Poland. To obtain microbial inoculum avoiding osmotic stress, foil samples with the remainders of soil attached to their surface were dipped in 0.9 % NaCl solution and gently shaken for 7 days. Then, the aseptic conditions were provided, and 1 ml of solution was inoculated into liquid medium Czapek-Dox for fungi used in the study of biodegradation of polymers by fungi of the *Aspergillus* genus (Hakkarinen and Albertsson 2004; Arutchelvi et al. 2008). The composition of the medium was modified, so that it did not contain carbon sources: NaNO_3_−2.0, KH_2_PO_4_−0.7, KCl−0.5, K_2_HPO_4_−0.3, MgSO_4_x7H_2_O−0.5, FeSO_4_x7H_2_O−0.01 (g/L). The culture was incubated at 27 °C for 14 days. Obtained biological material was proliferated at the next stage of the research. Six Petri dishes with 2.5 % LB medium solidified with 1.5 % agar were inoculated with 0.25 ml of isolated culture and incubated for 7 days at 27 °C. The microorganisms were inoculated into two flasks in the liquid modified Czapek-Dox medium (Konduri *et al*. 2010) and the modified Davis Minimal Broth medium: (NH_4_)_2_SO_4_−1, Na_2_HPO_4_−8.83, MgSO_4_−0.1, KH_2_PO_4_−2, NaH_2_PO_4_−1.15 (g/L) (Satlewal *et al.* 2008). The sole carbon source for microorganisms were sterilized HDPE foil fragments suspended in the medium. Immediately, the optical density OD_650_ was measured, and the cultures were incubated for 48 h with shaking 120 rpm (Satlewal *et al.* 2008) at 37 °C. OD_650_ was measured again, and the streaking plates on 2.5 % LB medium solidified with 1.5 % agar were prepared in order to obtain pure colonies for identification. Liquid cultures were incubated for 14 days at 27 °C. Biodegradation of HDPE samples was carried out in liquid Davis Minimal Broth medium in 4 flasks (assays 1–4) of 250-ml capacity. In 2 flasks (1, 2 assays), cultures were cultivated with 100 ml of modified medium without glucose and in the rest ones with 100 ml of medium with 30 % of the recommended by the producer (Sigma-Aldrich) glucose content quantity: (NH_4_)_2_SO_4_−1, Na_2_HPO_4_−8.83, MgSO_4_−0.1, KH_2_PO_4_−2, NaH_2_PO_4_−1.15, glucose−0.3 (g/1000 ml). Addition of glucose, as a simple organic carbon source, was to accelerate the development of microbial populations (Satlewal *et al.* 2008). The medium was inoculated with bacteria consortium, and sterile polyethylene foil samples were suspended in. Immediately, OD_650_ was measured, which was then repeated every 48 h up to the start of the film samples collecting for structural analysis, that is, for 20 days. The culture was incubated at 27 °C for 50 days, when HDPE samples to FTIR analysis were collected and then carried on up to 150th day when the last samples for weight control and SEM were collected.

### Identification of bacteria

DNA was extracted from cultures on agar plates using DNeasy Plant Mini Kit (Qiagen, Germany) and subjected to amplification of 16S ribosomal genes. PCR conditions were as follows: 30 cycles with primer annealing temperature set to 54 °C, reaction mixture (total 25 μl): contained 12.5 μl 2 × Green Master Mix (Fermentas, Lithuania), 1 μl of each primer (10 pmol) 8 F and 1492R (Turner *et al*. 1999), 9.5 μl water and 1 μl of DNA sample. PCR products were purified with the Wizard PCR kit (Promega, USA) and then cycle sequenced with ABI BigDye Terminator ver. 3.1 (Applied Biosystems, USA). Obtained sequence was subjected to GenBank under accession no. KP793145. BLAST (Altschul *et al*. 1990) was used to search the GenBank for the most similar sequences. Sequences with the highest similarity values and some other sequences representing other bacteria from GenBank were selected for further analysis. Data set was aligned using ClustalW (Thompson *et al*. 1994), then analyzed with MEGA5 (Tamura *et al*. 2011). Obtained sequence was identified as *Achromobacter xylosoxidans* (*Betaproteobacteria* class) and named PE-1 (GenBank). There are few studies reporting application of different strains of this gram-negative bacterium for degradation of hazardous substances, such as endosulfan (Li *et al*. 2008) or its resistance on high copper concentrations (Ng *et al*. 2012), which could be perspective for application of this bacteria for bioremediation in the broad sense.

### Polyethylene foil samples

Samples of polyethylene subjected to microbial activity were prepared by sectioning into 2 × 2-mm square-shaped pieces of HDPE film. These samples were cut out from easily available, new clean packaging bags. The foil was made of pure polyethylene according to information given by the producer on the packaging, which was confirmed by comparative analysis with the model HDPE FTIR spectra from Aldrich Collection of FT-IR Spectra Edition I database. All samples were disinfected with 70 % (*v/v*) solution of EtOH air-dried and weight. The control assay was prepared. The same pieces of HDPE foil were disinfected in 70 % (*v/v*) solution of EtOH, air-dried, dipped in the liquid Davis Minimal Broth, and stored under the same conditions of temperature and lighting, as the 1–4 assays, but without contact with microorganisms. Samples subjected to microbial activity were collected every 4 days starting from the 20th day of incubation to 50th day of incubation, in order to control the trend of chemical changes of the HDPE by FTIR analysis. The samples for weight control and SEM were collected after 150 days of incubation. After collecting *A. xylosoxidans* treated and untreated samples, each of them has been disinfected in a solution of 70 % (*v/v*) EtOH to remove the bacteria that colonized the foil film and then dried. The prepared samples were subjected to FTIR analysis. The spectra were recorded with a spectrometer Brüker Alpha-P with ATR head in the range from 400 to 4000 cm^−1^ and processed with Opus 6.5 software. Resolution of the measurement was set to 2 cm^−1^. The identity of the investigated sample of HDPE was confirmed by comparison with the spectrum from spectral library—Aldrich Collection of FT-IR Spectra Edition I. The structural changes of polymer were detected by SEM with parameters: Carl Zeiss camera (EVO MA 15) electron microscope, Carl Zeiss SmartSEM V05.04.00 software, and 20 kV filament current. Before observation, the dried samples were sputtered and coated, for ca. 5 min under an argon atmosphere, with gold.

## Results and discussion

### Culture growth kinetics control

**Fig. 1:**
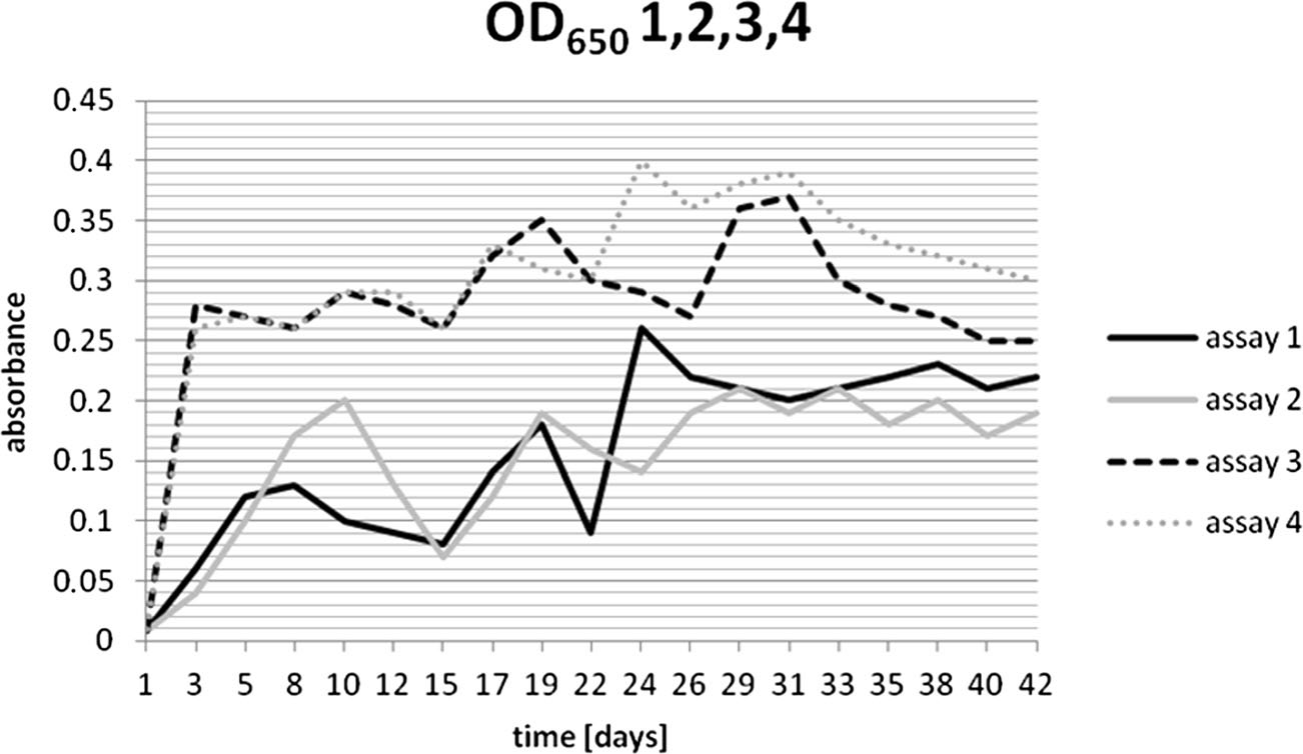
OD_650_ values for modified medium Davis Minimal Broth 1, 2 without glucose and 3, 4 supplemented with glucose assays

Monitoring of changes of OD_650_ between the culture growing in Czapek-Dox medium and the culture with Davis Minimal Broth medium allowed to obtain results indicating better growth of microbial population in Davis Minimal Broth medium. After 14 days of incubation, the bacteria population was higher in the medium Davis Minimal Broth (OD_650_ = 0.320) as compared to the population size in the medium Czapek-Dox (OD_650_ = 0.228). This result determined the choice of that medium for further studies. In assays 3 and 4, addition of glucose was significant for the growth of bacteria (Fig. 1). In these cultures containing glucose, the observed values of OD_650_ were higher, than in case of the assays 1 and 2 (Fig. 1) with no glucose.

**Fig. 2:**
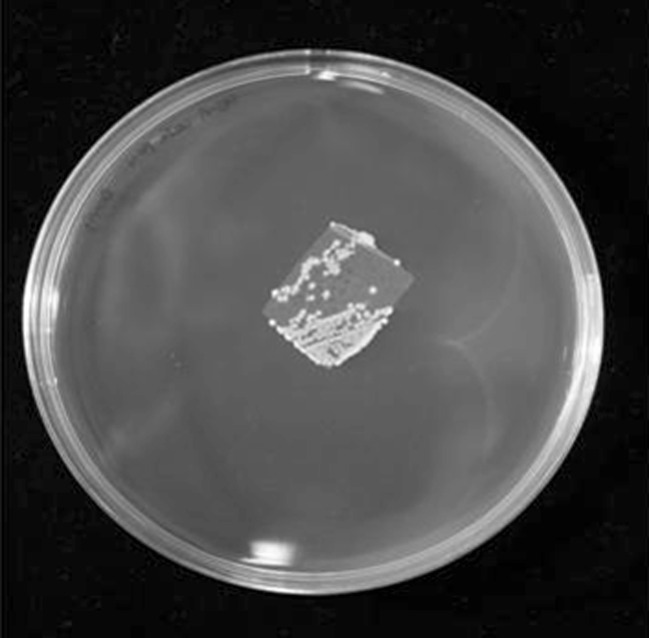
HDPE foil film colonized with *A. xylosoxidans* on Davis Minimal Broth without glucose (HDPE as sole source of carbon) 3 days after bacteria inoculation to the medium

Quite good growth of *A. xylosoxidans* was also observed on Davis Minimal Broth agar plates (1, 2 assays) with the HDPE as the sole carbon source in the medium. The colonization of HDPE foil by bacteria was detectable after 1-day incubation. Proliferation of bacteria on the HDPE foil was successful while entire experiment time (Fig. 2). The similar effect was observed in liquid cultures with HDPE as sole carbon source. The film of bacteria covering foil samples was also clearly visible.

Although quite good growth of A. *xylosoxidans* was observed both in liquid and agar media with HDPE as sole source of carbon, OD_650_ measurements (Fig. 1) indicate that the population of microorganisms effectively increased in the cultures with the addition of glucose. In the initial stage of incubation, OD_650_ was increasing for all of the analyzed samples (assays 1–4), and this growth was close to logarithmic. After about 10 days of incubation, there was a decrease in absorbance observed within all assays. After about 22–24 days of incubation, the population stabilized, and after 40 days, began to decrease, going in the death phase (Fig. 1). Within cultures supplemented with glucose (assays 3 and 4) in the initial incubation phase population, growth is observed too. Since the fifth day, all the cultures reached a stabilization phase, and the absorbance value was between the range of 0.243 to 0.424 a.u. These values are higher on average by 0.1 a.u. in comparison to assays without glucose (Fig. 1). HDPE samples were collected from the 20th day of the experiment simultaneously to the moment of population stabilization.

### Effect of *Achromobacter xylosoxidans* on chemical structure of HDPE samples

**Fig. 3:**
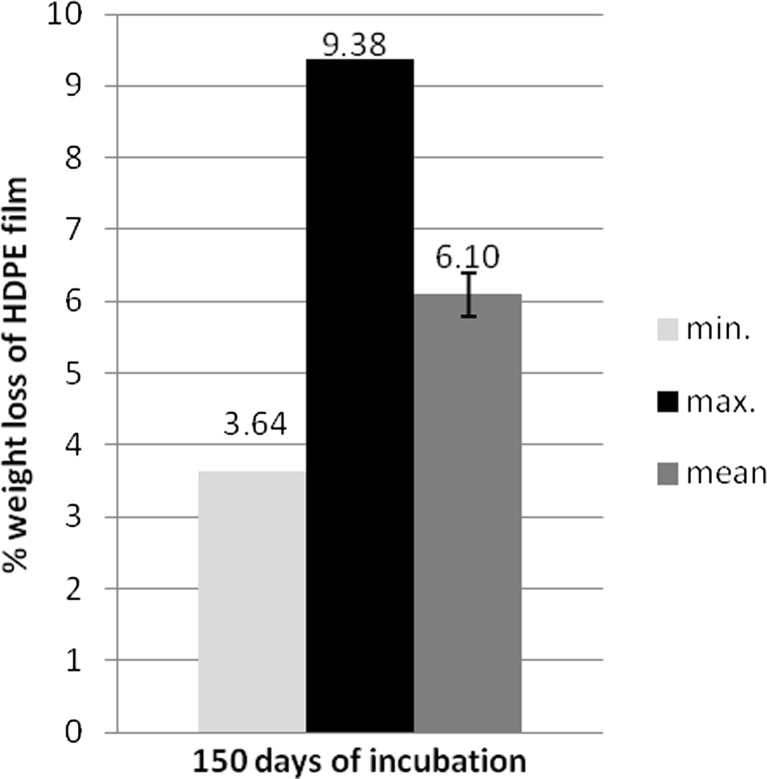
Percentage mass reduction of HDPE film after 150 days of incubation in modified Davis Minimal Broth medium

There are different factors indicating initiation of degradation process, e.g., harming chemical structure, demonstrated by depletion or creating new bonds, increase of hydroplilicity, or weight decrease (Tokiwa *et al.* 2009). The analysis of the mass of HDPE samples cultured in medium free of carbon except of HDPE demonstrated the loss of the residual weight. That was not observed in case of untreated samples. The percentage range of the mass decrease was between 3.64 and 9.38 % and 6.10 ± 0.13 % on average (Fig. 3). On the other hand, it should be mentioned that mass measurement of HDPE in the initial stage of decomposition of the polymer is not useful at all for the degradation study, because the sample weight loss is partly compensated by oxygen atoms introduced into the material in oxygenation processes (Czop and Biegańska 2012). To verify obtained results, FTIR and SEM methods were additionally applied. FTIR method is method is commonly and successfully applied both for biotic and abiotic HDPE degradation analyses (Corrales *et al*. 2002; Sanchez and Allen 2011; Erbetta *et al*. 2014; Immanuel *et al*. 2014).

FTIR measurement results are presented as vibrational-rotational spectra in Microsoft Office Excel 2010. The energy of vibrational- rotational is expressed as the dependence the amount of energy absorbed (absorbance) on the wave number (cm^−1^). The representatives of spectra of the foil samples from 1 to 4 assays, collected every 4 days for 24 days, beginning on the 20th day of the experiment, are presented. Table 1 presents the system of samples marking.

**Table 1:** System of labeling foil samples used to FTIR analysis

	Assay	Sample number						
		Day 20	Day 24	Day 28	Day 32	Day 36	Day 40	Day 44
Medium without glucose	1	1	5	9	13	17	21	25
	2	2	6	10	14	18	22	26
Medium with glucose	3	3	7	11	15	19	23	27
	4	4	8	12	16	20	24	28

**Fig. 4:**
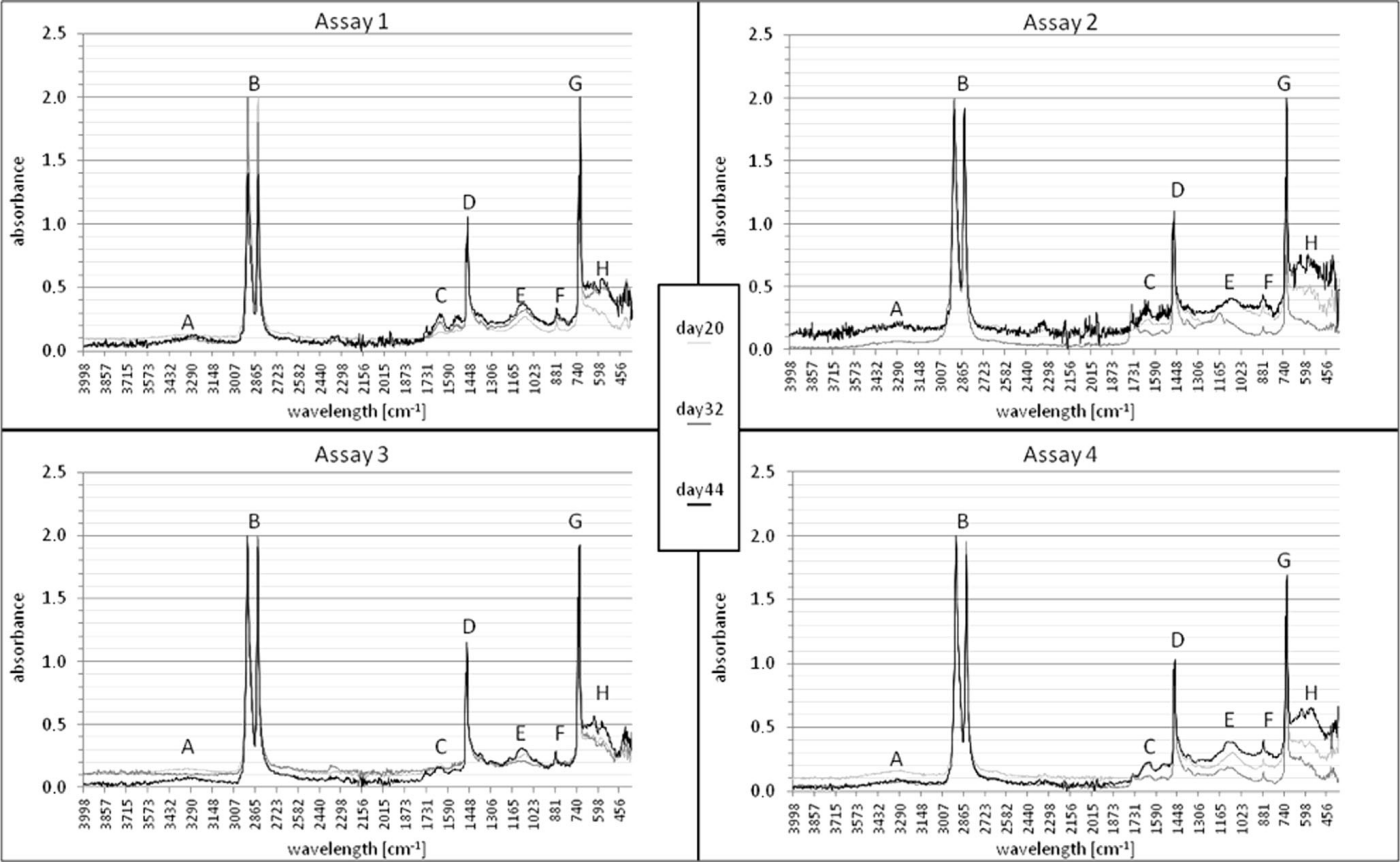
Spectra of samples collected from 1 and 2 assays without glucose and 3, 4 assays supplemented with glucose. Origin of bands: **a** −OH group, **b** −C-H group, **c** >C = O group, **d** −C-H group, **e** −C-O-O group, **f**, **h** >CH_2_ skeletal vibrations, **g** >CH_2_ group

Figure 4 presents the combinations of the spectra received by study of samples degraded in the medium Davis MB without glucose or any other carbon source (assays 1 and 2) and containing 30 % of recommended glucose content (assays 3 and 4). The only potential extra source of organic carbon in 1 and 2 assays, except to polyethylene scraps, might be the remains of microorganisms formed naturally, as a result of necrosis of the specimens. This process is the final phase of the liquid culture growth, which is determined by concentration of the toxic metabolites or when at least one necessary component of the medium is depleted (Schlegel 2003). Full degradation of the persistent plastic like HDPE is a long process. Therefore, for evaluation, the possibility of plastic degradation by bacteria FTIR technique was used.

The spectra showed clearly polyethylene chemical changes over time of the experiment, in all cases. In the range of approximately 3600–3200 cm^−1^ (A, Fig. 4) is the appropriate band -OH group, which may be part of the carboxyl group. The presence of the hydroxyl group provides an oxidization polyethylene chain, then attachment of a hydrogen atom and acidification of the medium. The oxidization influences on the properties of polyethylene, giving their surface more adhesive and hydrophilic. It should be mentioned, that the acidification might be the result of presence of microorganisms metabolites. This -OH band was observed particularly in the spectra of samples collected 36 and 40 days of the experiment (data not shown), and also 44 days of experiment (44. day presented on Fig. 4). The bands in the range of approximately 3000–2840 cm^−1^ (B, Fig. 4) corresponding to the -CH bonds remain unchanged, as it is a basic part of the molecule and is characteristic of aliphatic hydrocarbons. The same origin have bands observed at 1450 cm^−1^ (D, Fig. 4) (Silverstein and Webster 2005). Particularly important are the bands occurred in the range of approximately 1730–1650 cm^−1^. This is a result of the presence of the carbonyl group -C = O. The group is formed by embodying an oxygen atom in the damaged structure of the polyethylene chain. Thus, already the first spectrum of the sample from the assay 1 (Fig. 4, assay 1, sample 1—day 20, C) shows a weak vibration of the carbonyl group, increasing with time of the experiment. Under vibration approximately 1150–1075 cm^−1^ (E, Fig. 4), a range ether bonds -C -O -C- is observed. This type of bond is the result of the embodying oxygen atom between the C-C bonds weakened due to the high electronegativity of oxygen and the affinity of oxygen to carbon. In terms of 900–735 and less than 700 cm^−1^ vibrations (F and H, respectively, Fig. 4), there are bands of skeletal vibrations. The higher and more intensive absorbance presented in the area, the weaker the structure of the test compound. This is due to the fact that the cleavage or forming new bonds, the conformation of polyethylene structure is changed. The structure became more loose, which reduces the energy level and enhances atoms vibrations (Silverstein and Webster 2005).

It was expected that the glucose, as a simple carbon source, will accelerate the cells proliferation and increase the number of microorganisms. As a result, degradation of HDPE will be intensified (Satlewal *et al.* 2008). Analysis of samples from the cultures with glucose (assays 3 and 4) presented changes in the structure of HDPE very similar to those that can be observed in the case of assays without glucose (assays 1, 2 Fig. 4). The addition of glucose in the medium seems to have no influence on efficiency of biodegradation.

**Fig. 5:**
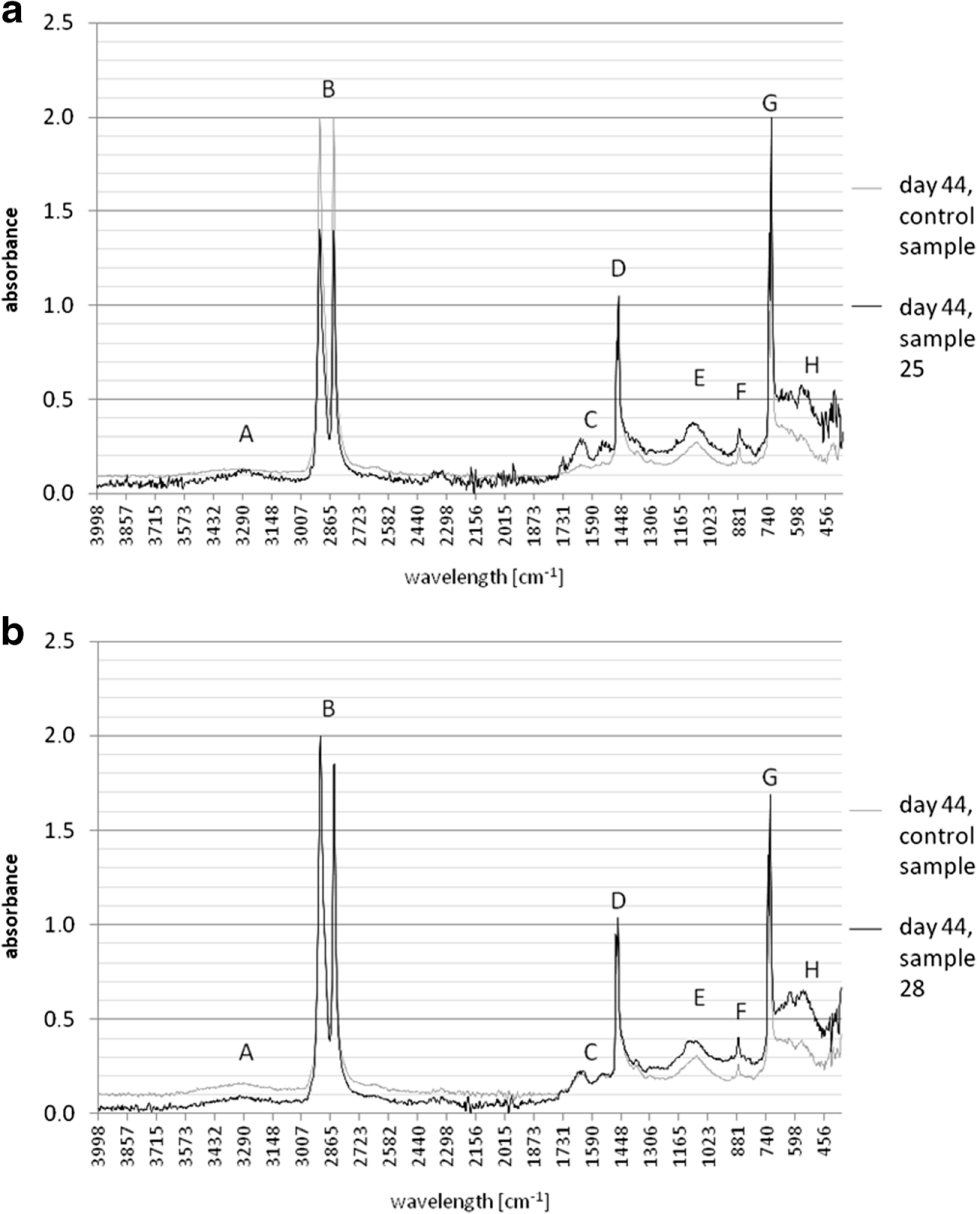
Final spectra of representative degraded samples in comparison to control samples, without glucose (sample 25 and control, a) and with addition of 30 % of recommended amount of glucose (sample 28 and control, b). Origin of bands: **a** −OH group, **b** −C-H group, **c** >C = O group, **d** −C-H group, **e** −C-O-O group, **f**, **h** >CH_2_ skeletal vibrations, **g** >CH_2_ group

The final spectrum of the sample 25 (assay 1, Fig. 5a) shows that the carbonyl band (appearing in the range of 1800–1635 cm^−1^, C, Fig. 5a) is presenting a greater intensity than the band of the same range of control sample. This correlation is also observed in the spectrum of the final stage of the experiment for all samples (data not presented), when after 44 days of degradation the carbonyl group, hydroxyl (3600–3200 cm^−1^, A, Fig. 5) and the ether group (about 1150 to 1070 cm^−1^, E, Fig. 5) bands have stronger intensity than the corresponding band of the control sample (samples 25, 28, Fig. 5). Skeletal vibrations (below 700 cm^−1^, H, Fig. 5) higher in the spectrum of the presented samples confirm a greater degree of degradation of the HDPE structure. In case of spectra of HDPE degraded in medium with glucose (Fig. 5b), bands of a carbonyl group, hydroxyl, and ether group do not vary sufficiently to demonstrate, that degradation of the samples by bacteria in medium supplemented with glucose is more progressive and intense than in case of samples degraded without glucose.

**Fig. 6:**
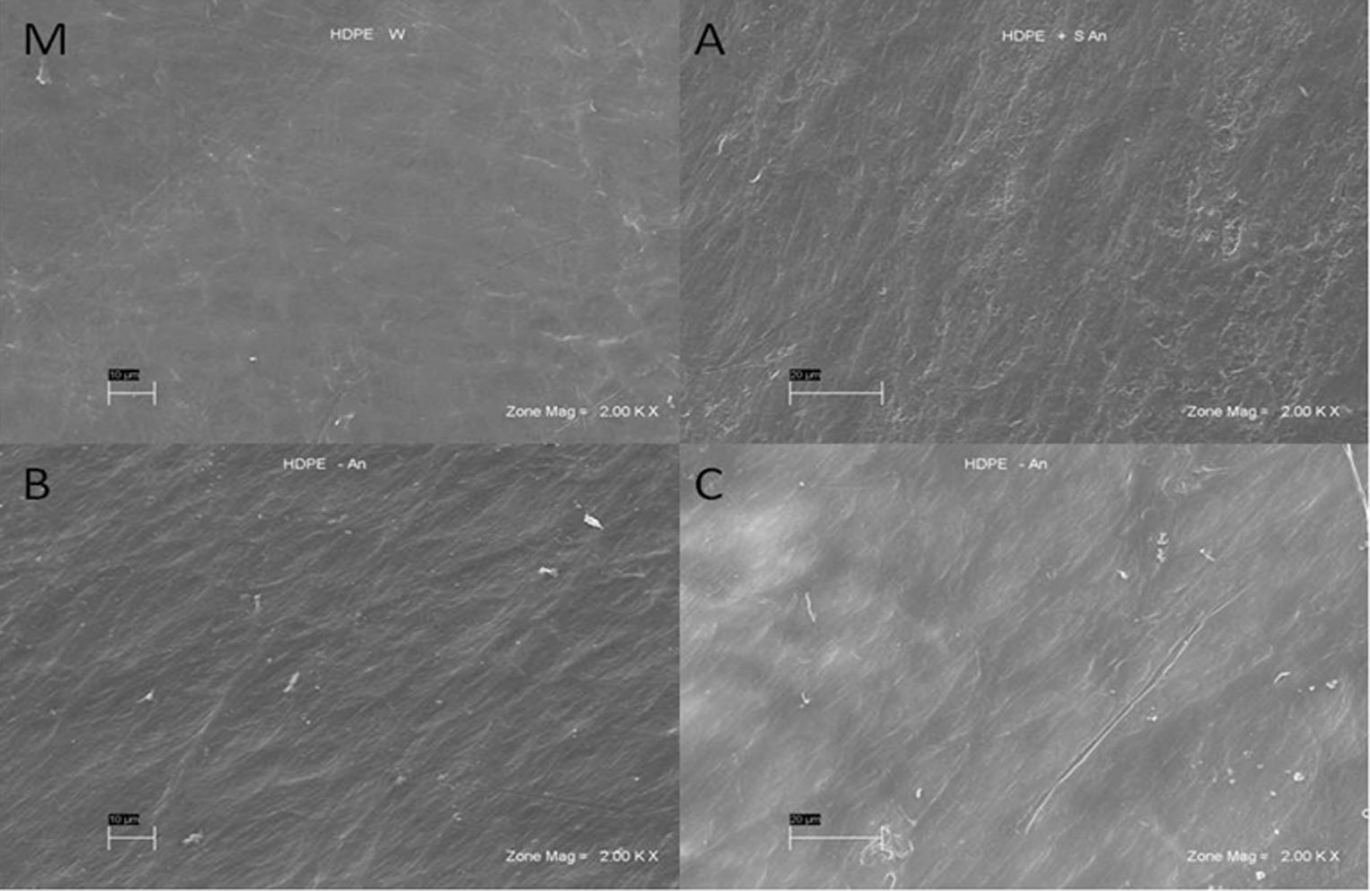
Representative SEM photographs (magnification 2000×) of untreated model virgin HDPE film sample (M) and subjected to *A. xylosoxidans* PE-1 activity (A-C) after 150 days of incubation

Modifications of the HDPE chemical structure treated by *A. xylosoxidans* PE-1 detected by FTIR technique were confirmed by SEM (Fig. 6). The photograph of bacteria-treated samples (Fig. 6a–c) demonstrate damages of the HDPE film surface, which is visibly rough in comparison to smooth surface of the sample not subjected to bacterial activity (Fig. 6M). Such disturbances were confirmed by analysis of 46 samples SEM photographs (magnifications 1000–10,000×, 50 % bacteria treated and 50 % untreated samples).

Spectral analysis of samples subjected to the activity of isolated *A. xylosoxidans* strain presented changes in the chemical structure, i.e., defragmentation of HDPE chains and the creation of new organic functional groups in the structure of polyethylene, as a result of microorganism activity. The increase in double bonds was also observed for degraded samples in several studies (Volke-Sepúlveda et al. 1999; Hakkarinen and Albertsson 2004). It is presented by the spectra for assay 4 (Fig. 4), where the single successive spectra are devoid of disturbances (caused by external factors during the test, such as variable humidity). Forming of new bands and their increasing intensity is associated with the duration of the culture and thereby the development of microbial population. A significant degradation of the structure of the foil samples takes place at the moment of population stabilization, about 20–22-day culture incubation (Fig. 1). The isolated strains of bacteria inhabiting the soil are likely to be able to adapt its metabolic cycles to grow on the surface, use carbon from the polyethylene eroding and thus weakening its structure (Bonhomme *et al.* 2003). FTIR measurements present the character of the degradation development and its progress (Fig. 4, Fig. 5). SEM photographs visibly confirm result of *A. xylosoxidans* PE-1 activity demonstrating damage of the samples surface (Fig. 6). Biodegradation of commercial HDPE proceeds slowly (Hakkarinen and Albertsson 2004), and low efficiency of this degradation process causes, that at this stage, it cannot be applied in industrial processes of utilization polyethylene waste, as HDPE structure changes emerged only at the molecular level. The molecular weight decrease significantly when polyethylene subjected to biodegradation is pre-oxidized. Abiotic factors like UV radiation or thermal oxidation increase the hydrophilicity of the polymer, which in turn increases the susceptibility of polyethylene to biodegradation (Albertsson and Bánhidi 1980; Chiellini *et al*. 2003; Hakkarinen and Albertsson 2004). It is also possible, that the degradation would proceed more efficiently with the cultures enriched with another strains of bacteria, like, e.g., *Arthrobacter sp.*, *Rhodococcus sp.*, *Pseudomonas sp.*, *Bacillus sp.* or fungi, e.g., *Aspergillus sp.*, *Penicillium sp.* (Albertsson et al. 1998; Chiellini *et al.* 2003; Sivan *et al.* 2006; Roy *et al*. 2008; Anwar et al. 2013; Immanuel *et al*. 2014; Arutchelvi *et al.* 2008;) used successfully in similar experiments.

## Conclusion

New strain of bacteria with ability the results to utilize polyethylene was identified. The results of this study revealing new ability of *A. xylosoxidans* PE-1 will contribute a significant information to research on PE biodegradation. Such property makes that bacteria another one in the variety of useful organisms reported to utilize HDPE in plenty of studies on polyethylene biodegradation, including fungi (Immanuel et al. 2014, Tokiwa *et al*. 2009), bacteria (Yamada-Onodera et al. 2001; Gilan *et al*. 2004; Hadad *et al*. 2005), and even worms and its endosymbiotic bacteria (Yang *et al*. 2014) and will allow to approach to work out of consortium degrading HDPE more efficiently.

## Abbreviations

ATR: Attenuated total reflectance
FTIR: Fourier transform infrared spectroscopy
HDPE: High-density polyethylene
PE: Polyethylene
SEM: Scanning electron microscope
